# Amplifying the Voices of Youth for Equity in Wellness and Technology Research: Reflections on the Midwest Youth Wellness Initiative on Technology (MYWIT) Youth Advisory Board

**DOI:** 10.2196/69013

**Published:** 2025-04-10

**Authors:** Linnea Laestadius, Fridarose Hamad, Leena Le, Rosemary Buchtel, Celeste Campos-Castillo

**Affiliations:** 1 Zilber College of Public Health, University of Wisconsin–Milwaukee, Milwaukee, WI, United States; 2 Department of Political Science, University of Wisconsin–Milwaukee, Milwaukee, WI, United States; 3 College of Natural Science, Michigan State University, East Lansing, MI, United States; 4 Department of Media & Information, Michigan State University, 404 Wilson Road, East Lansing, MI, 48824, United States, 1 (517) 355-3410

**Keywords:** advisory boards, adolescents, social media, qualitative research, community engagement

## Abstract

Incorporating youth perspectives into health research can enhance quality, relevance, and ethics while also providing youth with mentorship, exposure to academic research, and professional development opportunities. This has led to a growing number of youth advisory boards (YABs). However, despite increased attention to YABs, the mentions of YABs remain low in the published research on youth and health. Furthermore, little published work has reflected on the importance of engaging with youth of color in YABs. This is critical both because of the perspectives and insight they bring to the research process and to help close the participation gap in extracurriculars among youth from racial and ethnic minoritized groups. To contribute to the literature on YABs and health equity, we offer an overview and reflection on the development and implementation of the Midwest Youth Wellness Initiative on Technology (MYWIT), a 1-week virtual, financially compensated summer YAB for youth of color aged 13 to 17 years centered on amplifying youth voices on questions related to digital technology and mental health. MYWIT youth advisors successfully codeveloped a novel research question and semistructured interview guide on the topic of navigating social media algorithms. The MYWIT process also highlighted the importance of youth compensation levels, scheduling, recruitment strategies, and overall resource constraints. We hope to encourage researchers to reflect on the value that even short duration YABs can add to the research process and how YABs can be structured to better recruit and support advisors who experience economic, institutional, and structural barriers to participation.

## Introduction

There has been an increasing recognition of the importance of incorporating youth perspectives into the research process to enhance quality, relevance, and ethics while also providing youth with mentorship, exposure to academic research, and professional development opportunities [1]. This has led to a growing number of youth advisory boards (YABs), also known as young people’s advisory groups or youth advisory committees, where youth are recruited to help shape one or more stages in the research process. Health-focused researchers in particular have sought to create YABs [1-4], in part due to increased mandates by funders for patient and provider involvement [5].

This also aligns with the promotion of human rights, as Article 12 of the United Nations Convention on the Rights of the Child (UNRC) states that children shall hold the right to freely express their views in all matters affecting them [6]. In 2021, the UNRC clarified that the right to expression also applies to decisions relating to the digital environment [7]. Although the United States has not ratified the Convention, it remains a critical reference point for ethical research and practice. Amplifying youth voices in research on digital technologies and well-being has become particularly pressing as a growing number of policymakers and public figures have called for regulating youth access to social media and internet-enabled smartphones. More research is critically needed in this area, both to unravel the complexity of current evidence on the mental health impacts of digital technologies and to identify strategies for protecting youth without it coming “at the expense of children’s other rights” such as freedom of expression, information, and assembly [7]. To ensure evidence-based government and platform policies that promote well-being and health equity, youth perspectives, particularly those of youth from vulnerable and minoritized groups, are essential to crafting research questions and methods that reflect the lived experiences of youth.

Despite increased attention to YABs, mentions of YABs remain strikingly low in the published research on youth and health [8]. Further, little published work has reflected on the importance of engaging with youth of color in YABs. This is critical both for the perspectives and insight they bring to the research process and to help close the gap between youth from racial and ethnic minoritized and racial and ethnic majority groups in participating in nonsports-related extracurriculars [9]. Specifically, Hispanic youth have relatively low levels of participation in nonsports-related extracurricular activities, and non-Hispanic Black youth have not increased their participation rate in nonsports-related extracurriculars at the same pace as other groups [10].

These disparities in participation matter not just to college and future earnings trajectories [10-12], but more directly to well-being and health outcomes. Youth who participate in nonsports-related extracurriculars are less likely to smoke tobacco, drink alcohol, or use marijuana [10]. Access to these benefits is uneven and difficult to access for those without financial resources. Cuts to public services and a shift to a “pay-to-play” model of extracurriculars skew access toward higher-income families [9,11]. Youth with college-educated parents are also more likely to participate in extracurricular activities [10]. Further, youth participation in the paid workforce has been shown to displace their own participation in extracurricular activities, which may further disadvantage lower youth with fewer economic resources [13]. YABs seeking to incorporate perspectives from youth of color must therefore consider these financial needs.

In short, (1) YABs can offer benefits to youth of color and the advancement of equity-focused research on technology and well-being, and (2) it is critical that YABs financially compensate youth of color for their time to facilitate their participation over competing demands and formally recognize their contributions. Collaborative research with members of the public, particularly with those from racial and ethnic minoritized groups, has a responsibility to center “questions of equity and justice” [14]. To contribute to the literature on YABs and health equity, we offer a brief overview and reflection on the development and implementation of a 1-week virtual, financially compensated summer YAB for youth of color aged 13 to 17 years residing in the US Midwest. We call the YAB the Midwest Youth Wellness Initiative on Technology (MYWIT). The MYWIT is a collaborative effort between faculty at Michigan State University and the University of Wisconsin-Milwaukee. We launched the MYWIT in 2022 via seed funding from the Technology and Adolescent Mental Wellness YAB Initiative at the University of Wisconsin-Madison to help meet the goal of amplifying the voices of youth from racial and ethnic minoritized communities in research on digital technologies and youth mental health. Together, we created a research protocol and a draft semi-structured interview guide, illustrating the value of youth perspectives even in the formative stages of health equity research.

## Youth Advisory Boards

YABs are closely related to youth participatory action research (YPAR), but generally stop short of allowing youth to cocreate and participate in all aspects of the research process and may lack the intention of “shifting power structures” inherent to the YPAR orientation [15]. While acknowledging this limitation, YABs offer an advantage in terms of logistical flexibility, allowing for more widespread adoption across a range of methodologies, time frames, and disciplines. They can be used to help develop a single research study or to provide insight and guidance on a broader research area or endeavor [1]. YABs thus have the potential to become a widely adopted tool in public health and medical research. The involvement of target populations in research can improve study feasibility and the usefulness of findings for practice [16,17]. Participating in the research process can also have benefits for youth above and beyond any financial compensation received, including positive outcomes related to leadership (eg, confidence and self-efficacy), academic/career (eg, time management, and oral and written communication), and social support (eg, sense of belonging and community) [2,18]. A YAB can collaborate on tasks such as generating or refining ideas, offering feedback on data collection tools, troubleshooting study challenges, and aiding in interpreting and disseminating findings [1,2,19]. Given the range of scope for YABs, guidelines have been proposed to ensure consistent reporting on how youth contribute to research [8].

However, YABs are not without challenges. Studies of YABs note several barriers to engaging with youth, including navigating ethics approvals, uncertainty about the value that youth could add, concerns about risk and the potential for harm to youth advisors, insufficient time and resources, lack of support from institutions, and recruitment challenges [20,21]. Prior work has also noted difficulties with scheduling, access to technology, attendance, and social dynamics such as a reluctance to share or the formation of cliques [2,19]. Youth may also experience anxiety about engaging with a process that is new to them [22]. Furthermore, YAB conveners must work to ensure that they provide advisors with meaningful experiences and that contributions are felt to be respected and valued rather than tokenistic, even if suggestions are not feasible within the current research environment due to discordance with institutional policies or a lack of funding [17,21,23]. These challenges also highlight the broader issue of “adultism,” which privileges the perspectives and norms of adults through bias against youth [24,25]. While YABs are designed to elevate the voices of youth, norms, and expectations may still lead to an environment where youth needs are not centered or their perspectives are not fully respected [17]. Finally, the lack of alignment between the institutional expectations placed on faculty and the extensive work needed to develop and implement a YAB also mean that the work of supporting a standing YAB may need to be taken on by academic staff [23], which necessities substantive funding or other forms of institutional support. These barriers are key to overcoming for YABs to fulfill their potential of working toward addressing health equity.

## The Midwest Youth Wellness Initiative on Technology (MYWIT)

### Aims

The MYWIT holds the goal of working toward health equity by amplifying the voices of adolescents aged 13 to 17 years who self-identify with communities of color around the Midwest region of the United States. Specifically, the MYWIT program aims to (1) diversify the voices heard within conversations about how technologies impact teen well-being, (2) encourage advisors to share their experiences with technologies and discover new research questions that have never been asked before, (3) train advisors in the scientific method so that they can contribute to shaping research studies and consider a research-related career, and (4) prepare advisors to be leaders by providing them with a platform and skill set to communicate their ideas with others.

### Format and Recruitment

In the first year of the MYWIT (ie, 2022), we operated using an academic year model, with online meetings every other month over the course of 12 months. Owing to challenges with scheduling and attendance, as also noted in a prior study [2], we opted to pilot a 1-week virtual summer camp model in 2023, meeting for 3 hours each morning on Zoom between July 17 and 21, 2023. Summer is also a time when extracurricular activities hold particular value due to summer learning loss [26]. A virtual format was selected due to the broad Midwestern catchment of the MYWIT, making a singular in-person meeting impossible. While virtual YABs can lead youth to feel less comfortable sharing their personal reflections and perspectives, prior YABs suggest that this barrier decreases over time as the advisors begin to feel more comfortable with each other [2]. Furthermore, online YABs eliminate the need to worry about how advisors will travel to meetings [1]. The objective was to offer youth an introduction to social science research methods and then support the advisors in collaboratively developing a research question and data collection tool to support future research. The Institutional Review Board at the University of Wisconsin-Milwaukee determined that the work did not qualify as human subject research. As compensation for their time, advisors received a US $100 honorarium if they attended all 5 sessions (with exceptions made for emergency situations). While contact information for a trusted adult was gathered in case of an emergency, the honorarium was distributed directly to each advisor via email in the form of two US $50 Amazon gift cards. All advisors were also offered the opportunity to coauthor this manuscript, which has been noted as a rare but important means of recognizing youth participation [17,27].

**Figure 1.**
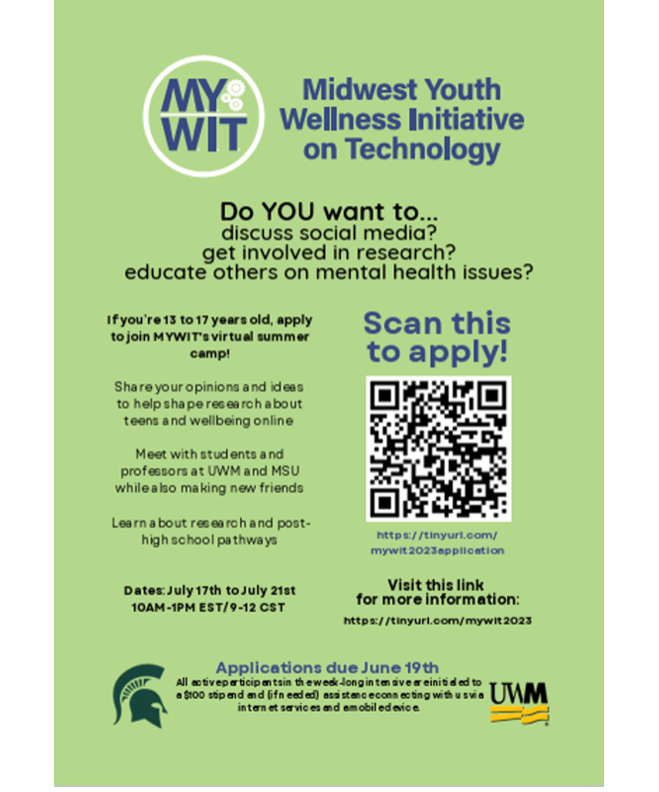
The Midwest Youth Wellness Initiative on Technology (MYWIT) recruitment flyer.

In addition to two mid-career faculty leads (one from sociology and one from public health) who served as codirectors, the MYWIT was supported by two undergraduate students (one from political science and one from public health) compensated through a university mechanism to support undergraduate research. All team members were female with a broad range of backgrounds (Latina, Asian, Middle Eastern, and European). In spring 2023, the undergraduate student mentors recruited eligible teens through the dissemination of a recruitment flyer (Figure 1) via personal contacts, outreach to local youth organizations throughout the Midwest, and through university staff that worked with high school recruitment and TRIO Upward Bound programs. Of 12 applications received, 4 youths (3 from Milwaukee, WI and 1 from Ann Arbor, MI) responded to emails and text messages to schedule informal interviews and complete emergency contact information. All advisors were between the ages of 16 and 17 years; 2 identified as male and 2 as female; 3 identified as Black or African-American and 1 as Asian and White. The number of advisors enrolled was lower than our initial target of 7-8. Additionally, no Hispanic youth enrolled as advisors. Notably, the cohort size was larger and more diverse in the first year of the program. It is not clear if the change for this cohort was due to the shift away from the academic year model as the recruitment strategy was similar for both cohorts. One participant indicated a need for technology resources and was provided with a university-owned tablet for the duration of the program. Having devices available and screening for need is critical since limited access to devices is a potential barrier to equitable participation for online YABs [2].

### Lesson Topics and Research Output

Faculty and student mentors collaborated on organizing the program through brainstorming sessions informed by prior experiences with teaching research methods and a suggested outline for how to structure each day created by ChatGPT 3.5. The team had prior experience tailoring research design to high-school students; both faculty leads had mentored incoming first-year college students and one of the student mentors had engaged in research with the codirectors in her first summer after high school. Each morning, we started with an icebreaker or game related to technology or wellness for advisors to build rapport and prepare them for the day’s activities and topics. We created Canva slides for lessons and used Google Jamboard and Padlet to engage youth advisors in brainstorming, feedback, and collaboration activities. Each day’s activities primed the advisors for the next day’s topic and equipped them with new concepts, terminology, and skills related to research. Camera use was encouraged but not required.

The topic for each day can be seen in Table 1. Lessons on research were paired with research output-oriented activities each day. To begin, we asked advisors to brainstorm research topics related to technology in their lives. On day 2, they developed the research question: “How do minority youth navigate social media algorithms to meet their needs (social and consumer needs)?” Specifically, advisors felt that social media algorithms often showed them products not aligned with their needs (eg, haircare products for White hair types) or that targeted them in ways that felt manipulative (eg, ads for colleges that feature diverse students, when most of the student body is White). While it is difficult to study algorithms directly due to their proprietary nature, prior research suggests that social media algorithms may either target or exclude users from racial and ethnic minoritized groups in discriminatory ways and may also privilege social media content from White creators, denying users and creators “equal access to services and opportunities” [28,29]. Thus, the question centered on how youth from racial and ethnic minoritized communities work to navigate social media algorithms to better find the products and communities that meet their needs. Although the undergraduate student mentors and faculty codirectors reflect a broad range of racial and ethnic backgrounds, we note that we lacked concordance with the Black or African-American majority of the advisors. While advisors spoke about racialized experiences on social media, this lack of concordance may have shaped interactions in ways that either encouraged or inhibited discussion about these topics [30].

**Table 1.** The Midwest Youth Wellness Initiative on Technology (MYWIT) summer programming, 2023.

MYWIT summer program	Lessons and activities	Research output from advisors
Day 1: Introduction to Mental Health and its Relationship to Technology	Introductions and icebreaker Interactive tutorial on current evidence and advisor perspectives on the pros and cons of social media for youth mental health Jamboard brainstorming session: “Now it’s your turn to be the researcher. What questions need to be asked? What needs to be explored?”	List of ideas for research studies about technology and youth well-being
Day 2: Research 101	Icebreaker Educational session on the scientific method, social science research, and research ethics Activity comparing social science research with natural science research Refinement of brainstorming from Day 1 to reach one shared research question Education session on common social science research and data collection methods Activity for picking a data collection method aligned with the research question	A single open-ended research question to guide the rest of the week: “How do minority youth navigate social media algorithms to meet their needs (social and consumer needs)?” A data collection approach and target population: Semistructured interviews Youth age 13 to 17 years All minoritized groups Social media users (any platform)
Day 3: Developing a Research Plan and Expert Panel	Icebreaker Education session on how to craft effective interview questions Padlet brainstorming session for interview questions to respond to research question Advisors led a mock interview with one of the undergraduate student mentors followed by question refinement Expert panel and question and answer sessions with technology and mental health entrepreneurs	List of ideas for semistructured interview questions
Day 4: Finalizing the Research Plan and Program Reflection	Icebreaker Final revisions to interview questions Jamboard brainstorming session on what advisors hope will change based on the study and how to recruit participants Padlet reflection on the MYWIT: What did you learn? What did you enjoy? What would you change?	Final list of semistructured interview questions List of study justifications and recruitment strategies Reflections and advice on the youth advisory board process
Day 5: Post Program and College Advice (short session)	Guidance on how to highlight the MYWIT participation on college applications and resumes Advice for how to participate in research as an undergraduate student Sharing of information on how to stay in touch and assessing interest in being involved in implementing the proposed study	List of advisors interested in continued work

On days 3 and 4, advisors designed a research protocol and semistructured interview guide that could be implemented in the future (Textbox 1). To provide additional professional development content, the MYWIT also featured an invited panel of entrepreneurs of color who work in the fields of technology and mental health and concluded with a session on how to translate their new research expertise into posthigh–school experiences. With the exception of this final day, which was led fully by the student mentors, each day had one of the codirectors present to support the advisors.

Textbox 1. **Interview questions developed by youth advisors on the topic of how youth from racial and ethnic minoritized groups navigate social media algorithms.**

1. What is your favorite app?
2. How is your social media feed different now than when you first joined?
3. What kinds of things have you done to personalize your social media feed? How has that worked for you?
4. What kinds of obstacles have you encountered when navigating social media?
5. How does social media affect your mental health?
6. What social media app do you use to get recommendations or inspiration for things you want to buy?
7. What social media app do you use to search for things you want to buy?
8. What kinds of products do you look for on social media?
9. What word or phrase would you use to search for these products?
10. How does it make you feel looking for products on social media that fit your needs?
11. Do you feel like it is hard to find products for you on social media? Why?
12. Have you ever felt excluded by an ad on social media?
13. Can you give an example of a time when you felt excluded by an ad on social media?
14. How do you feel about social media advertisements targeting minorities?
15. What app do you use to search for communities online?
16. How do you use that app to interact with people?
17. How did you find those communities on that app?
18. Do you feel like it is hard to find communities for you online?
19. Have you ever felt left out from communities on social media because of your culture? Can you give an example?
20. How does it make you feel looking for communities on social media to fit your needs?
21. Do you post on social media?
22. What kinds of hashtags or buzzwords do you use to get your posts seen by the people they are meant for?
23. What would you change about the app to make it more inclusive for your community?
24. What would you change about the app to make navigating the app easier?

## Experiences and Recommendations

### Lessons Learned

At the end of the week, everyone reflected on the process and their recommendations via three questions on Padlet: (1) What did you learn? (2) What did you enjoy and what worked well? (3) What would you change? Additionally, advisors were asked to share further brief reflections via email following the final day. Below, we present the synthesized reflections across the groups (codirectors, student mentors, and advisors). Table 2 provides additional detail on key lessons learned.

**Table 2.** Key lessons from the Midwest Youth Wellness Initiative on Technology (MYWIT).

Topic	Key lessons
Recruitment	Recognize that recruitment is the most difficult task. Budget extra time for recruitment. Significantly overenroll in anticipation of attrition before the program begins so that the final cohort is larger.^b^ Cold calling youth-serving organizations was not effective. Adopt rolling application review until the YAB^a^ begins. Reduce the amount of time between the application going live and the start of the YAB^b^ Holding advisor interviews can help assess the level of interest and commitment to attend. Inviting advisors’ friends to participate did not prove to be a very effective recruitment strategy. Most advisors came from organizations that researchers and youth mentors had direct connections with or from a posting on the Michigan State University’s website. Outreach within team member networks is a promising means of recruitment.
Programming and Engagement	Interactive icebreakers were important to encourage participation from advisors. From the beginning, have a clear attendance policy (cameras on, how many days they can miss, etc). Change the scope and duration of the program to allow for more in-depth work. Mentors and Codirectors suggested shorter program length with longer days, while Advisors suggested a 2-week program with shorter days.^b^ The most reliable communication to ensure daily attendance was texting. Emails seemed to work less frequently. Family or financial commitments may prevent advisors from giving their full attention during sessions. Incentives should be informed by what advisors might make in hourly employment during the duration of the program. Additional resources are needed to support study development and implementation with the same set of advisors. Consider in-person meetings or ways to make online meetings more engaging.^b^ Despite obstacles, youth advisors have novel and valuable insights into research goals and processes.

a YAB: youth advisory board.

b Indicates items based in part on advisor feedback

### Advisors

Each of the advisors shared what they learned, what they enjoyed, and what they would change about the experience. Responses highlighted learning how to craft research questions and collect relevant data. Advisors also noted that they valued learning that other youth shared their concerns about digital technologies and enjoyed learning about social media and health. One advisor noted that learning how to develop interview questions was interesting because it taught them how to gather information without making assumptions about research participants. Another stated that they found it a fun, low-stakes entry into scientific research, allowing them to build up confidence for future opportunities. Advisors reported enjoying getting to craft a question that they care about and the panel discussion with Milwaukee, WI technology experts on relevant careers. Some feedback they provided for improvement included having a shorter application time span so that more youth would join and not forget about the program in the middle of the summer, enrolling more youth, and extending the program over 2 weeks and having shorter days instead. Additionally, two advisors indicated that in-person meetings would have been helpful. One explained that they felt the online format made sense for the time, but that having future programs in-person would facilitate conversations, as there were frequent silences during meetings that could be attributed to many of the advisors not turning on their cameras, leading to black screens with no face showing.

### Undergraduate Student Mentors

Student mentors reported learning as peer mentors to the youth advisors, specifically enhancing their community engagement and mentorship experience for future careers. Both were nervous about explaining the program in a way that would be interesting and exciting to the advisors but enjoyed the challenge of practicing how to communicate clearly. Seeing how well the youth advisors engaged made the student mentors feel confident in their own communication growth. The students learned that if there are clear explanations and expectations of the youth advisors, they will feel empowered and motivated to participate, which will enhance the likelihood that the products contribute toward health equity. Student mentors also learned the importance of adjusting communication from emails to phone calls or texts to send reminders to advisors and keep them engaged. Recommendations for change include a later start time for youth to be more engaged and recruiting more advisors to make it a more dynamic experience.

### Faculty Codirectors

Codirectors had initially been worried about the low enrollment numbers, which persisted despite proactive efforts to recruit advisors, and the financial incentive offered. Ultimately, the 4 advisors generated valuable insights into research goals and processes, with the momentum of the 1-week schedule potentially offsetting the small group size. It is possible that the smaller-than-anticipated cohort size helped to achieve this positive outcome, as the literature on virtual focus groups indicates that smaller sizes (4 to 6) are preferred to achieve engagement and foster trust in the absence of traditional face-to-face communication [31].

However, not all advisors were able to participate equally during the week. One advisor joined from their place of summer employment and was thus not able to consistently make use of video and audio functions on Zoom. Developing clear guidelines in advance for what counts as full participation is essential. Furthermore, incentives may need to be increased to better reflect what youth could earn in hourly employment during the duration of the program. The loan of a tablet computer to an advisor also posed complexities in terms of institutional policies, with the university requiring that the student sign a document that they would cover the cost of the tablet if it was lost. Accordingly, a tablet that was valued less than the incentive for participation was chosen to avoid creating a potential hardship in the event it was lost or damaged. Considering ways to overcome economic, institutional, and structural barriers to participation is critical for any program seeking to prioritize the voices of youth from minoritized and marginalized communities as a step toward centering equity in technology and wellness research.

Furthermore, while the MYWIT is out of necessity virtual due its broad catchment area, others may wish to explore in-person YABs. Although past works suggest that in-person YABs also struggle with attrition [3,32], initial recruitment may be easier if youth are seeking a more traditional and immersive summer experience. We may also reconsider our policy on camera use being optional during online meetings to help foster a greater sense of community among the advisors. Camera use can be an equity concern, due to camera access, internet speed, and a desire to obscure the residential environment. In addition to the provision of devices when needed, one approach to consider might be to encourage the use of virtual backgrounds to allow cameras to be on, while still maintaining greater privacy [31].

Despite these challenges, the codirectors were extremely pleased with the rapid pace at which advisors picked up key research concepts, the insights advisors offered, and their passion for technology and youth wellness. Undergraduate mentor support was also pivotal for establishing rapport with the advisors. For example, undergraduate mentors helped to troubleshoot communication by suggesting the use of text messages rather than email. While this may appear to be an obvious choice, institutional norms for communication with college students via email (particularly at public institutions, where email is considered a public record) can obscure the actual communication preferences of youth and young adults. This serves as an indication of the critical importance of reflecting on and working to shift “adultism” when engaging with YAB work [24].

The research question that the advisors developed was one that the team had not previously considered, and the proposed interview questions were critical to fully understanding how the advisors operationalized the concept of navigating social media algorithms. Accordingly, the YAB provided significant value to the research process through the development of unique research questions and methods informed by the lived experiences of the advisors and their perceptions of social media, which is key for working toward health equity. Overall, the objective of training advisors in the scientific method so that they can contribute to shaping research studies was achieved and the building blocks for a viable future study were created. Going forward, the MYWIT would benefit from additional financial and time resources to support (1) rapidly developing and launching the resulting study idea, and (2) allowing advisors to participate in actual study implementation.

## Next Steps

Sufficient resources are essential to support YAB work. For the MYWIT, we hope to obtain grant funding to support translating the advisor-developed project into a full-scale study, including resources to support youth, either through a YAB to offer guidance at key stages or a YPAR approach to more formally engage youth as partners throughout. A previous study on YABs indicates that advisors, with support and training, can excel at qualitative data collection and analysis [32]. The inability to move directly into the implementation of the ideas should not be seen as an indication that the YAB failed to generate valuable ideas and guidance, but rather an artifact of a small initial budget and the institutional prioritization of grant-funded work. Insufficient financial resources have been noted as one of the most common barriers to engaging youth in the research process [21]. Further reflective of the ways in which the organizational work needed to support a YAB is often misaligned with university expectations for faculty [23], we also hope to obtain the funding needed to hire not just student mentors but also academic staff to support future YAB work. This would also help create stability and continuity in the YAB over time [23]. Finally, we hope to draw on the recruitment lessons outlined in Table 2 to increase youth compensation and adopt new recruitment strategies to create a larger pool of advisors. This will also help to ensure engagement remains robust even if an advisor faces unexpected constraints that prevent their full participation.

## Conclusions

Through sharing our experiences with a compensated YAB for youth of color in the US Midwest, our hope is to not just encourage researchers to reflect on the value that even short-duration YABs can add to the research process, but also how YABs can be structured to better support advisors from racial and ethnic minoritized groups. While prior work has stressed the potential value of YABs and how they might be organized [2,3,8], this second question has received less attention. Sharing lessons, both in terms of what worked and what did not, is critical for identifying how best to leverage the value of YABs in shaping research on technology and health equity.

## Abbreviations

MYWIT: Midwest Youth Wellness Initiative on Technology
UNRC: United Nations Convention on the Rights of the Child
YAB: youth advisory board
YPAR: youth participatory action research

## References

[1] Moreno MA, Jolliff A, Kerr B (2021). Youth advisory boards: perspectives and processes. J Adolesc Health.

[2] Abraham O, Rosenberger CA, Poku VO (2023). Implementing a youth advisory board to inform adolescent health and medication safety research. Res Social Adm Pharm.

[3] Orellana M, Valdez-Soto M, Brockman TA (2021). Creating a pediatric advisory board for engaging youth in pediatric health research: a case study. J Clin Transl Sci.

[4] Tsang VWL, Chew SY, Junker AK (2020). Facilitators and barriers to the training and maintenance of young persons’ advisory groups (YPAGs). Int J Pediatr Adolesc Med.

[5] McCoy MS, Warsh J, Rand L, Parker M, Sheehan M (2019). Patient and public involvement: Two sides of the same coin or different coins altogether?. Bioethics.

[6] United Nations (1989). Convention on the Rights of the Child.

[7] Livingstone S, Sylwander KR (2025). There is no right age! The search for age-appropriate ways to support children’s digital lives and rights. J Child Media.

[8] Sellars E, Pavarini G, Michelson D, Creswell C, Fazel M (2021). Young people’s advisory groups in health research: scoping review and mapping of practices. Arch Dis Child.

[9] Heath RD, Anderson C, Turner AC, Payne CM (2022). Extracurricular activities and disadvantaged youth: a complicated—but promising—story. Urban Educ (Beverly Hills Calif).

[10] Meier A, Hartmann BS, Larson R (2018). A quarter century of participation in school-based extracurricular activities: inequalities by race, class, gender and age?. J Youth Adolesc.

[11] Snellman K, Silva JM, Frederick CB, Putnam RD (2015). The engagement gap. Ann Am Acad Pol Soc Sci.

[12] Lipscomb S (2007). Secondary school extracurricular involvement and academic achievement: a fixed effects approach. Econ Educ Rev.

[13] Crispin LM, Kofoed M (2019). Does time to work limit time to play?: Estimating a time allocation model for high school students by household socioeconomic status. Contemp Econ Policy.

[14] Fiske A, Prainsack B, Buyx A (2019). Meeting the needs of underserved populations: setting the agenda for more inclusive citizen science of medicine. J Med Ethics.

[15] Ozer EJ, Abraczinskas M, Duarte C (2020). Youth participatory approaches and health equity: conceptualization and integrative review. Am J Community Psychol.

[16] Arumugam A, Phillips LR, Moore A (2023). Patient and public involvement in research: a review of practical resources for young investigators. BMC Rheumatol.

[17] Hawke LD, Relihan J, Miller J (2018). Engaging youth in research planning, design and execution: practical recommendations for researchers. Health Expect.

[18] Anyon Y, Bender K, Kennedy H, Dechants J (2018). A systematic review of youth participatory action research (YPAR) in the United States: methodologies, youth outcomes, and future directions. Health Educ Behav.

[19] Flicker S, Guta A, Larkin J (2010). Survey design from the ground up: collaboratively creating the Toronto teen survey. Health Promot Pract.

[20] Wadman R, Williams AJ, Brown K, Nielsen E (2019). Supported and valued? A survey of early career researchers’ experiences and perceptions of youth and adult involvement in mental health, self-harm and suicide research. Res Involv Engagem.

[21] Faithfull S, Brophy L, Pennell K, Simmons MB (2019). Barriers and enablers to meaningful youth participation in mental health research: qualitative interviews with youth mental health researchers. J Ment Health.

[22] Mawn L, Welsh P, Kirkpatrick L, Webster LAD, Stain HJ (2016). Getting it right! Enhancing youth involvement in mental health research. Health Expect.

[23] Hohenemser LK, Marshall BD (2002). Utilizing a youth development framework to establish and maintain a youth advisory committee. Health Promot Pract.

[24] Bell J (1995). Understanding Adultism - A Major Obstacle to Developing Positive Youth-Adult Relationships.

[25] Florio E, Caso L, Castelli I (2020). The Adultcentrism Scale in the educational relationship: Instrument development and preliminary validation. New Ideas Psychol.

[26] Atteberry A, McEachin A (2021). School’s out: the role of summers in understanding achievement disparities. Am Educ Res J.

[27] Fadiran B, Lee J, Lemminger J, Jolliff A (2021). How our technology use changed in 2020: perspectives from three youths. JMIR Ment Health.

[28] Ali M, Sapiezynski P, Bogen M, Korolova A, Mislove A, Rieke A (2019). Discrimination through optimization. Proc ACM Hum-Comput Interact.

[29] Amarikwa M (2023). Social media platforms’ reckoning: the harmful impact of TikTok’s algorithm on people of color. SSRN Journal.

[30] Kerstetter K (2012). Insider, outsider, or somewhere between: the impact of insider, outsider, or somewhere between: the impact of researchers’ identities on the community-based research process. J Rural Soc Sci.

[31] Lathen L, Laestadius L (2021). Reflections on online focus group research with low socio-economic status African American adults during COVID-19. Int J Qual Methods.

[32] Livingood WC, Monticalvo D, Bernhardt JM (2017). Engaging adolescents through participatory and qualitative research methods to develop a digital communication intervention to reduce adolescent obesity. Health Educ Behav.

